# Follicular Thyroid Carcinoma Metastasis to the Retromolar Trigone: A Case Report

**DOI:** 10.7759/cureus.103245

**Published:** 2026-02-08

**Authors:** Homood M Almutairy, Hassan Alshurafa, Meshal Alsaedi, Yazieed M Albarrak, Rawan M Alahmadi, Fareed R AlGhamdi, Alyaa S Al Mutairy, Yazeed A Alkhalifah

**Affiliations:** 1 Otolaryngology - Head and Neck Surgery, Prince Sultan Military Medical City, Riyadh, SAU; 2 College of Medicine, King Saud University Medical City, Riyadh, SAU; 3 College of Medicine, Taibah University, Medina, SAU

**Keywords:** follicular thyroid carcinoma, oral metastasis, retromolar trigone, thyroid cancer metastasis, thyroid malignancy

## Abstract

Follicular thyroid carcinoma (FTC) may occasionally present with metastatic disease at atypical oral sites. This report describes a case of FTC involving the retromolar trigone. A 48-year-old woman with a history of FTC treated a decade earlier with total thyroidectomy and radioactive iodine therapy presented with a two-month history of a mass in the left retromolar region. Histopathological analysis of an incisional biopsy confirmed a metastatic well-differentiated follicular thyroid carcinoma. A conservative surgical approach involving left-sided modified radical neck dissection, followed by radioactive iodine management, caused complete resolution of the patient’s retromolar lesion. Follow-up over six months showed no evidence of recurrence. This case highlights a rare presentation of FTC metastasizing to the retromolar trigone. It underscores the importance of maintaining a high index of suspicion for metastatic disease in unusual oral lesions in patients with a history of thyroid cancer.

## Introduction

Follicular thyroid carcinoma (FTC) arises from the follicular cells of the thyroid and is recognized as the second most common type of thyroid cancer [1]. The diagnosis of FTC is made postoperatively through histopathological examination, as distinguishing it from benign follicular lesions preoperatively can be challenging. Unlike papillary carcinoma, which tends to spread through the lymphatic system, FTC more commonly metastasizes via the bloodstream [2]. As a consequence of this hematogenous pattern, distant metastases most frequently involve the lungs and skeleton [3]. Skeletal metastases typically affect the axial skeleton (particularly the spine and pelvis), while craniofacial involvement, including the skull and mandible, is uncommon but well described [4]. In reported facial skeleton metastases from FTC, the mandible is among the most frequently involved sites [5]. The retromolar trigone is an anatomically complex oral subsite posterior to the last mandibular molar, closely related to the mandible and masticator space [6]. Importantly, FTC metastases may present synchronously or as delayed events years to decades after initial treatment, underscoring the need for long-term vigilance when new skeletal or craniofacial lesions arise in patients with a history of FTC [7]. Oral and maxillofacial metastases from FTC are exceedingly rare, accounting for only 1%-2% of all oral and maxillofacial malignancies [5]. FTC metastasizing to the oral cavity is rare; accurate diagnosis calls for keen awareness and high suspicion. Imaging modalities such as positron emission tomography (PET) scans, CT, and MRI are key in identifying and evaluating metastatic disease [8]. Given the rarity of FTC metastasis to the oral cavity and maxillofacial skeleton, the aim of this case report is to raise awareness that delayed craniofacial/oral metastasis can occur years after initial treatment, may mimic primary oral pathology, and warrants long-term vigilance with timely radiographic evaluation when new oral or craniofacial lesions develop.

This case was previously presented as a poster at the 15th EROC Global Forum 2026 (January 15-17, 2026, Dubai, United Arab Emirates).

## Case presentation

A 48-year-old Saudi woman with a known history of follicular thyroid carcinoma for more than 10 years presented with a progressively enlarging left retromolar mass of two months’ duration, without constitutional symptoms or smoking history.

Her past medical history was significant for FTC only (diagnosed in 2013), for which she underwent total thyroidectomy and radioactive iodine therapy in 2014. She had no other relevant comorbid systemic illnesses documented at presentation. Dental history was unremarkable, with no recent dental extraction, odontogenic infection, maxillofacial trauma, or prior similar oral lesions. In 2023, after being in remission since 2014, she started developing clavicular pain with supraclavicular swelling for six months. Upon evaluation, metastatic FTC to the sternum and anterior lower neck was discovered. She was treated with sternal tumor resection and primary closure, and adjuvant radioactive iodine. Routine post-ablation whole-body scan follow-up showed a soft tissue lesion, about 1.2 cm in diameter, in the right neck, representing a metastatic right level 3 cervical lymph node. After that, multiple osseous lesions with high radioiodine activity were noted in the left mandible.

On physical examination, an approximately 3 cm left retromolar mass was noted with the fullness of the gingival mucosa over the affected area. It was associated with mild pain. There was no numbness, trismus, or bleeding. There was no palpable cervical lymphadenopathy, heart palpitation, or heat or cold intolerance. There was an increase in weight, with normal appetite and regular sleep.

**Figure 1:**
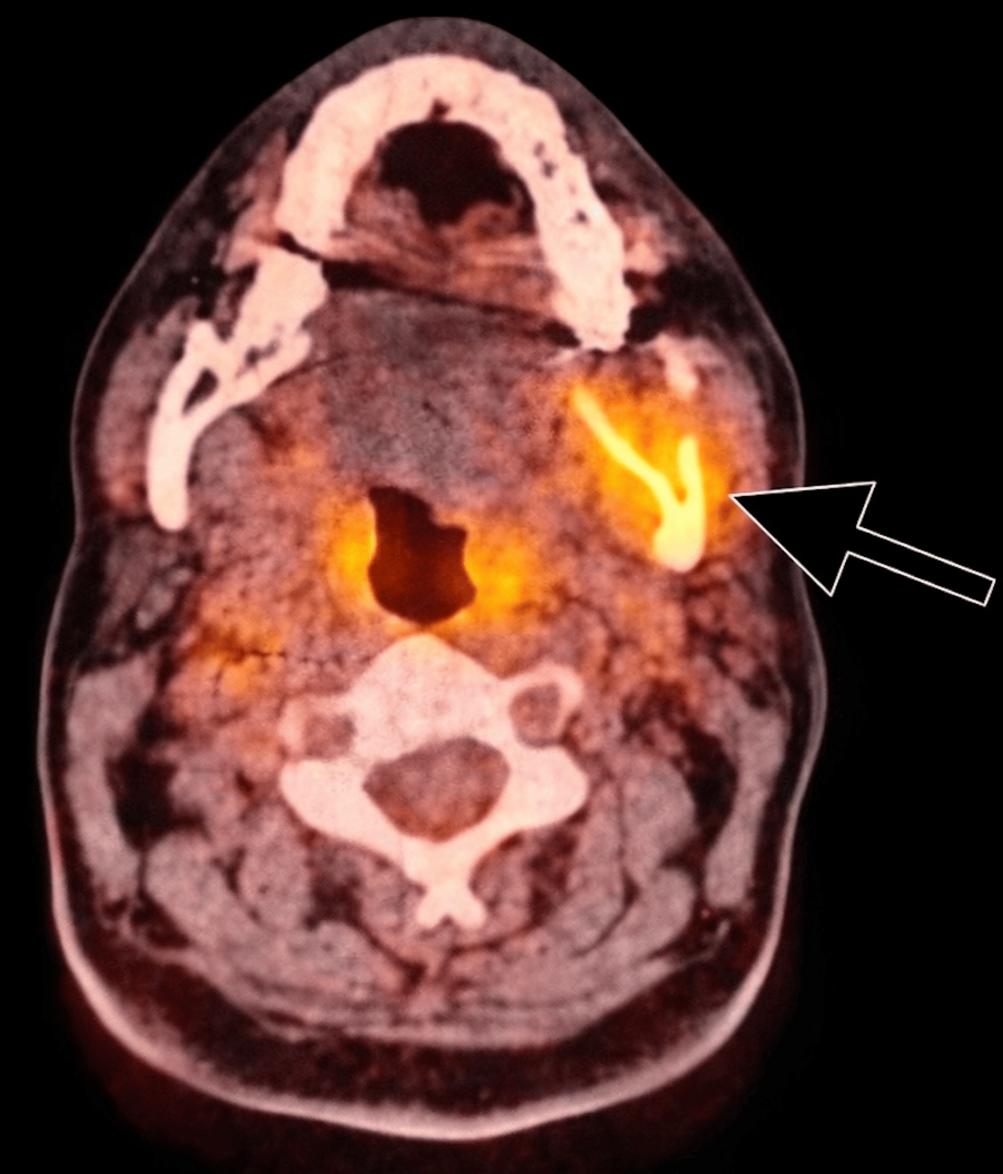
PET/CT imaging showing hypermetabolic activity in the left mandibular region PET: positron emission tomography/computed tomography

**Figure 2:**
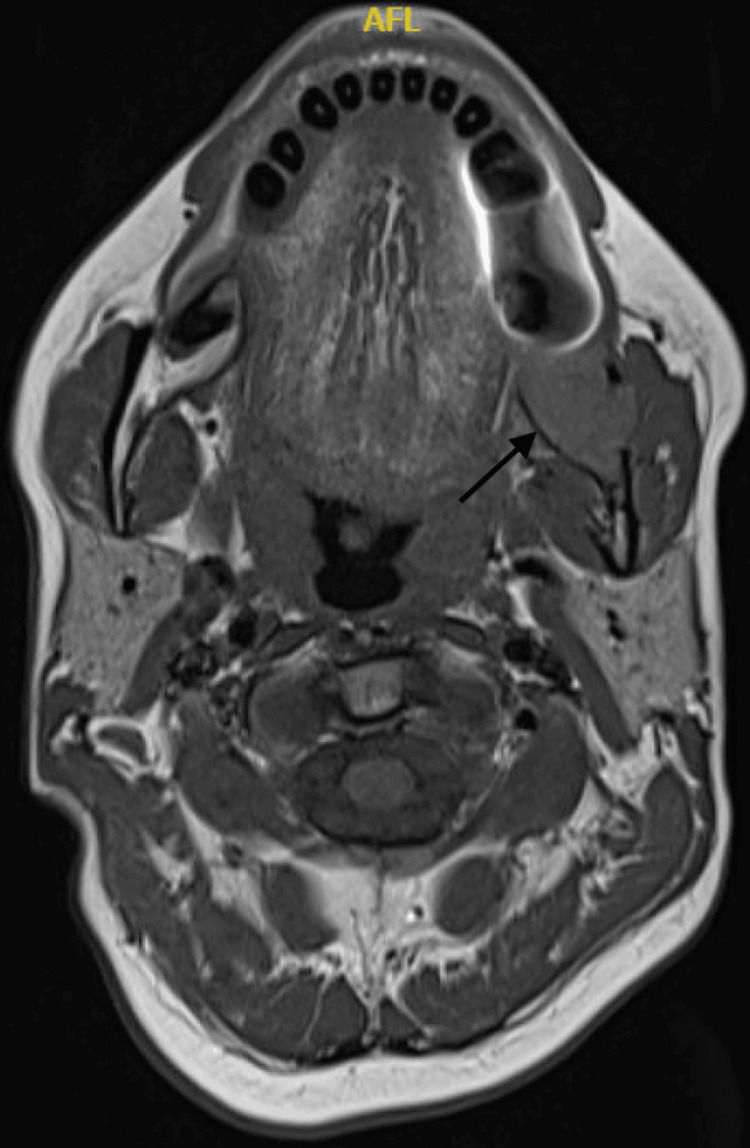
MRI of the left mandibular angle lesion with metastatic characteristics

A thyroglobulin test revealed significantly high levels, reaching 2157 ng/mL (reference range: 1-30 ng/mL). An incisional biopsy confirmed metastatic follicular type, well-differentiated thyroid carcinoma. Immunohistochemical staining was positive for TTF-1 and PAX-8, supporting thyroid origin. A CT scan revealed a stable osseous metastatic lesion involving the left mandibular angle. Multiple stable metastatic cervical lymph nodes were noted bilaterally, with the largest node in the right level 3 measuring approximately 0.9 cm and the left level 2 node measuring 1.0 x 0.7 cm. PET-CT imaging demonstrated focal hypermetabolic activity (SUV max 7.7) related to the surgical suture at the left upper retrosternal area, suggesting residual active disease or recurrence. The left posterior mandible showed an expansile lytic lesion with interval progression in size and metabolic activity (SUV max 10.4 compared to 3.6 in a prior scan), as shown in Figure 1. MRI showed an expansile lesion at the left mandibular angle involving the mandibular canal, consistent with metastatic disease. Multiple bilateral cervical and supraclavicular lymph nodes were identified, with the right level 3/4 lymph nodes appearing inseparable from the anterior aspect of the internal jugular vein, raising suspicion for possible tumor invasion. No intracranial metastasis was detected, as shown in Figure 2.

Treatment planning was multifaceted. We structured the treatment plan with both clinical judgment and the patient’s personal preferences in mind. After reviewing the options, she chose a conservative path, proceeding with a left-sided modified radical neck dissection. This was followed by radioactive iodine therapy to address any residual disease. The goal was to balance effective treatment and minimize surgical invasiveness. Postoperative histopathology confirmed the presence of metastatic follicular thyroid carcinoma, which supported the initial diagnosis and validated the course of treatment. At her sixth-month follow-up, she was doing very well. The retromolar lesion had resolved entirely, and there were no signs of persistent or recurrent disease. Her thyroglobulin levels had also returned to normal, suggesting a strong response to therapy. She remains in stable condition, with no evidence of recurrence so far, a reassuring outcome that reflects our approach’s effectiveness.

## Discussion

Follicular thyroid carcinoma spreads hematogenously, mainly to the lungs and bones. Oral metastasis from follicular thyroid carcinoma is exceedingly rare [5]. Within this spectrum, metastasis to the mandibular region is uncommon but has been reported; however, metastasis to the retromolar trigone appears to be unprecedented, as a thorough PubMed search yielded no documented cases of FTC metastasis specifically to this anatomical region. The rarity of this metastatic site presents significant diagnostic challenges. The retromolar trigone is an anatomically complex region, and metastatic disease in this area is rarely suspected in patients without a primary head and neck malignancy [6].

Several reports in the literature describe mandibular metastases of FTC presenting as asymptomatic swelling or mimicking odontogenic lesions, osteosarcoma, or primary jaw malignancies [5,9]. In our case, the lesion initially appeared clinically as a localized retromolar trigone soft-tissue mass; mandibular involvement (including the mandibular angle/canal region) was identified later on radiologic staging. Histopathological confirmation remains the diagnostic gold standard. Differentiating thyroid-origin metastatic lesions from primary maxillofacial malignancies relies on specific immunohistochemical markers, including thyroglobulin, TTF-1, and PAX-8 [10]. In this case, the positive TTF-1 and PAX-8 results supported a thyroid origin for the metastatic lesion and confirmed the diagnosis.

The treatment approach for FTC metastases in the maxillofacial region relies on tumor burden assessment, iodine uptake patterns, and systemic disease progression. Although surgical resection remains a viable option, some FTC metastases are resistant to radioactive iodine, requiring alternative strategies such as external beam radiotherapy (EBRT) or systemic therapies (e.g., tyrosine kinase inhibitors, or TKIs) [11]. In our case, due to extensive systemic disease, a conservative surgical approach followed by systemic radioactive iodine therapy was chosen, balancing disease control with the patient’s overall condition and preference. We acknowledge that the six-month follow-up period reflects only an early response to therapy, and longer term surveillance is necessary to adequately assess treatment efficacy and disease control. Due to the unusual location and absence of prior case reports, management of FTC metastasis to the retromolar trigone requires an individualized multidisciplinary approach integrating Otolaryngology - Head and Neck Surgery, Nuclear Medicine, Oncology, and Maxillofacial Surgery expertise.

## Conclusions

This case illustrates an unusual oral presentation of metastatic follicular thyroid carcinoma involving the retromolar region, confirmed on histopathology and managed with a combination of surgical and radioactive iodine-based therapy. Recognition of metastatic disease in atypical oral lesions is important, particularly in patients with a prior history of differentiated thyroid carcinoma, as it can alter diagnostic pathways and management. Reporting such presentations adds to the clinical spectrum of FTC and may help inform future diagnostic and therapeutic decision-making.
